# Far-red light modulates grapevine growth by increasing leaf photosynthesis efficiency and triggering organ-specific transcriptome remodelling: Author

**DOI:** 10.1186/s12870-024-04870-7

**Published:** 2024-03-15

**Authors:** Junhua Kong, Yan Zhao, Peige Fan, Yongjian Wang, Xiaobo Xu, Lijun Wang, Shaohua Li, Wei Duan, Zhenchang Liang, Zhanwu Dai

**Affiliations:** 1 State Key Laboratory of Plant Diversity and Specialty Crops, Beijing Key Laboratory of Grape Sciences and Enology, Institute of Botany, Chinese Academy of Sciences, Beijing, 100093 China; 2 China National Botanical Garden, Beijing, 100093 China; 3 University of Chinese Academy of Sciences, Beijing, 100049 China

**Keywords:** Grape plantlet, Photoassimilate, Photosynthetically active radiation, Shorter wavelength light

## Abstract

**Background:**

Growing evidence demonstrates that the synergistic interaction of far-red light with shorter wavelength lights could evidently improve the photosynthesis efficiency of multiple species. However, whether/how far-red light affects sink organs and consequently modulates the source‒sink relationships are largely unknown.

**Results:**

Here, equal intensities of white and far-red lights were added to natural light for grape plantlets to investigate the effects of far-red light supplementation on grapevine growth and carbon assimilate allocation, as well as to reveal the underlying mechanisms, through physiological and transcriptomic analysis. The results showed that additional far-red light increased stem length and carbohydrate contents in multiple organs and decreased leaf area, specific leaf weight and dry weight of leaves in comparison with their counterparts grown under white light. Compared to white light, the maximum net photosynthetic rate of the leaves was increased by 31.72% by far-red light supplementation, indicating that far-red light indeed elevated the photosynthesis efficiency of grapes. Transcriptome analysis revealed that leaves were most responsive to far-red light, followed by sink organs, including stems and roots. Genes related to light signaling and carbon metabolites were tightly correlated with variations in the aforementioned physiological traits. In particular, *VvLHCB1* is involved in light harvesting and restoring the balance of photosystem I and photosystem II excitation, and *VvCOP1* and *VvPIF3*, which regulate light signal transduction, were upregulated under far-red conditions. In addition, the transcript abundances of the sugar transporter-encoding genes *VvSWEET1* and *VvSWEET3* and the carbon metabolite-encoding genes *VvG6PD*, *VvSUS7* and *VvPGAM* varied in line with the change in sugar content.

**Conclusions:**

This study showed that far-red light synergistically functioning with white light has a beneficial effect on grape photosystem activity and is able to differentially affect the growth of sink organs, providing evidence for the possible addition of far-red light to the wavelength range of photosynthetically active radiation (PAR).

**Supplementary Information:**

The online version contains supplementary material available at 10.1186/s12870-024-04870-7.

## Background

Photosynthesis is one of the most critical biochemical processes in the world, in which plants, algae and cyanobacteria utilize solar energy to synthesize organic material and release oxygen, supplying food and oxygen for most life on Earth. Therefore, improving photosynthetic efficiency to increase crop yields is an essential measure to cope with future global food and security issues. Photon absorption by phytochromes determines the efficiency of photosynthesis in a wavelength-dependent manner. The sunlight spectrum consists of light with various wavelengths, including ultraviolet light (UV, 10–380 nm), visible light (400–700 nm) and far-red light (FR, 700–800 nm). It is well known that chlorophyll-a shows maximum absorption at 430 and 662 nm, chlorophyll-b at 457 and 646 nm, and carotene at 460 and 490 nm [1–3]. Therefore, it has been believed that only visible light can be used for photosynthesis, and the 400–700 nm photons are also known as photosynthetically active radiation (PAR) [4]. In contrast, far-red light was initially considered useless for photosynthesis, as plants could not carry out photosynthesis when far-red light was applied alone [5]. Nevertheless, when plants are exposed to longer- and shorter-wavelength light simultaneously, the net photosynthesis is far higher than the sum of the two lights applied independently. This phenomenon is known as the Emerson enhancement effect [6], and further investigations suggested that far-red light contributes to photosynthesis through a synergistic effect with shorter wavelength lights [7]. Moreover, chlorophyll-d and chlorophyll-f have been found in cyanobacteria, which can absorb photons at wavelengths of 700–750 nm for photosynthesis [8, 9]. However, to date, chlorophyll-d and -f have not yet been detected in higher plants, and the molecular mechanisms of the ‘Emerson enhancement effect’ of far-red remain largely unexplored.

The plasticity of morphological and physiological traits allows plants to make adjustments when sensing light fluctuations in their environments. Generally, plants grown in far-red conditions often present typical shade avoidance syndromes, such as stem elongation [10]. For example, irradiation of pine seedlings with far-red light (low R/FR) resulted in an increase in stem length and a decrease in total dry weight [11]. Conversely, filtering far-red solar light could inhibit internode growth and leaf area enlargement in chrysanthemum and bell pepper [12]. In addition, the low R/FR treatment could increase the content of carbohydrates in many species, including tomato [13, 14], turnip (*Brassica rapa* L. ) [15], onion [16] and cucumber [17, 18]. Similarly, supplemental far-red light could significantly increase the fresh weight, dry weight and stem length of lettuce [19]. Concurrently, an increase in leaf area was often accompanied, which may increase the light capture area and consequently increase the accumulation of carbon assimilates [19, 20]. Furthermore, when geranium and snapdragon plants were grown under the same photon flux density, the total leaf area, stem length and net photosynthesis of the whole plant linearly increased as the proportion of far-red light increased [21]. In tomato, additional far-red increased dry mass partitioning to fruit, resulting in a significant increase in fruit yield [13, 14], indicating that far-red also participates in regulating photoassimilate partitioning. Moreover, far-red light was found to inhibit lateral root development by modulating gibberellin transport [22]. More recently, Zhen and collaborators systematically studied the effects of the addition, substitution and filtering of far-red light on photosynthesis [5, 7, 23–25]. Regardless of whether plants were grown in the greenhouse or field, all these treatments consistently demonstrated that far-red light synergistically reacted with traditional photosynthetic photons (400–700 nm) and improved photosynthetic efficiency at three scales: single leaf, canopy and ecosystem [5, 7, 23–25]. In addition, far-red light can improve the photosynthetic efficiency in a variety of plants, including C3 and C4 plants, indicating that the promotion effect of far-red light on photosynthesis is rather common in higher plants [7]. However, the effect of far-red light supplementation on grapevine, a perennial plant, has not been investigated. Chen and Blankenship [8] noted that if plants could utilize far-red photons between 700 and 750 nm, the number of photosynthetic photons could increase by 19%, resulting in a significant increase in crop yield. Overall, far-red light is believed to contribute to regulating plant growth and photosynthesis with beneficial effects on biomass production. Besides, appropriate light recipe is demonstrated to be conducive to cultivate high-quality grape plantlets and accelerate plant growth in the greenhouse [26].

The photosynthesis process involves a series of reactions carried out in photosystem I (PSI) and photosystem II (PSII). Previous studies have demonstrated that longwavelength far-red light and shortwavelength light preferentially excite PSI and PSII, respectively [27, 28]. However, light-capturing complexes LHCB1 and LHCB2 shuttled between the two photosystems to ensure the balance of the excitation of the two photosystems, in turn resulting in high efficiency of plant photosynthesis [29]. The far-red light increases photosynthetic efficiency most likely by activating photosystem I synergistically with shortwavelength photons. However, it is not clear whether the effect of far-red light is restricted in the leaves or could be systematically propagated to other sink organs, such as stems and roots; what are the transcriptome remodelling processes triggered by far-red light to orchestrate the source and sink organs for improving photosynthesis efficiency?

The present study aimed to determine the effect of far-red light supplementation on grape plantlet growth, photosynthesis efficiency and photoassimilate partitioning among organs, as well as to clarify the underlying regulatory mechanisms. To this end, plant growth indicators, photosynthesis parameters and carbon assimilate contents in different organs were monitored for plants grown under far-red supplementation. Subsequently, transcriptome analysis was conducted with different organs (leaves, stems and roots) to elucidate the comprehensive regulation of far-red light signals among organs. Correlation network analysis integrating physiological traits and the transcriptome was conducted to identify key genes related to light signals and carbon metabolites. This study provides novel insights into why far-red light could improve photosynthesis efficiency and regulate carbon assimilate allocation in grapevine.

## Methods

### Plant material and treatments

**Fig. 1:**
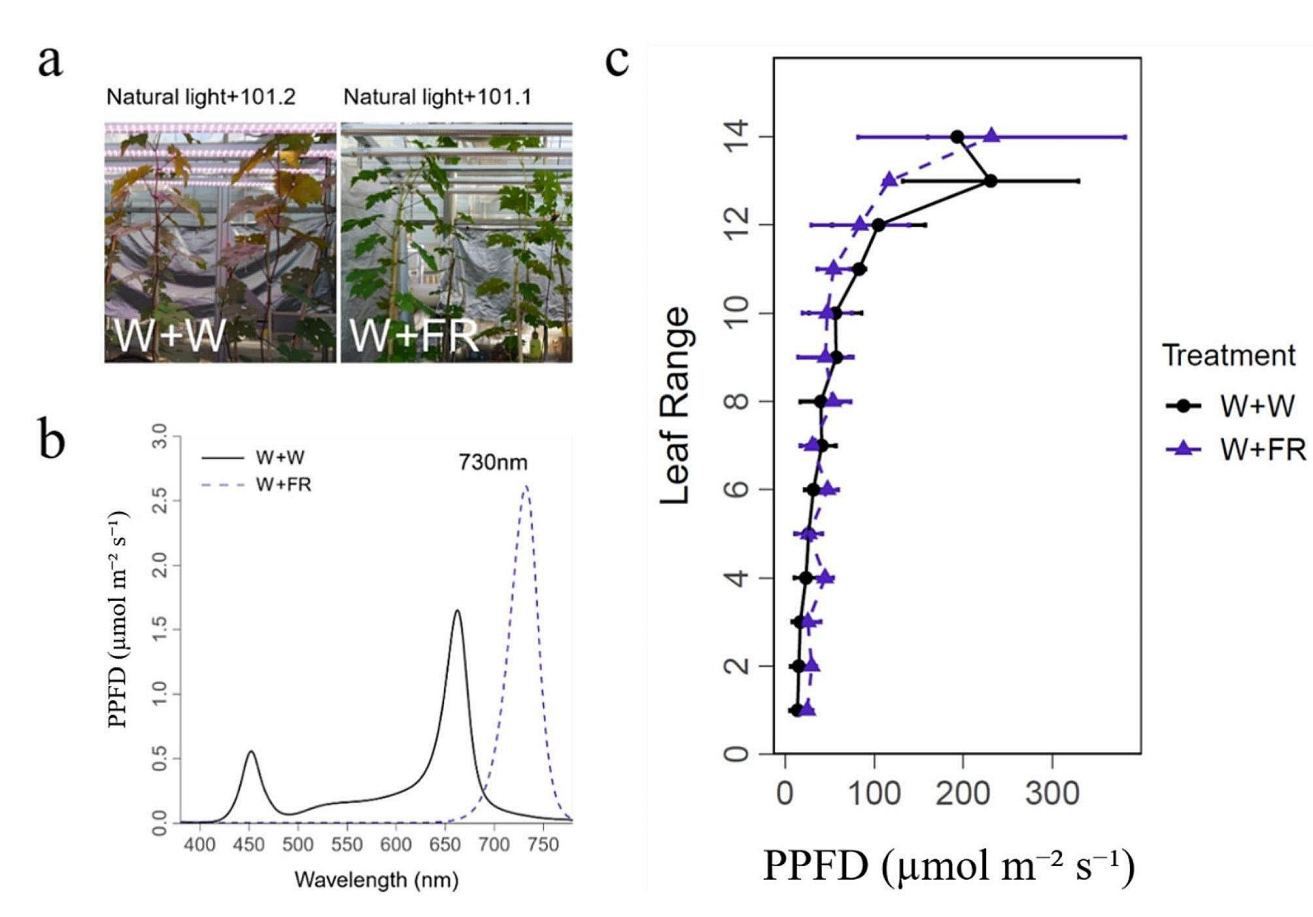
Environmental conditions and LED spectra for additional far-red light treatments. W + W and W + FR denote white light supplementation in natural light and far-red light supplementation in natural light at a light intensity of 101 µmol m^− 2^ s^− 1^, respectively. The LED spectra of white and far-red light resources were measured with a light spectrometer (LI-180, LI-COR) at 40 cm below the lamps during the night, and the peak wavelength for the far-red LED was at 730 nm (**a**, **b**). Light intensity at the original leaf position under different supplemental light treatments was measured at 25 days after treatment (**c**)

The experiment was conducted with 1-year-old grapevine self-rooted cuttings (*Vitis vinifera* cv. Cabernet Sauvignon, from the same clone #169) grown in a glass greenhouse, at the Institute of Botany, Chinese Academy of Sciences, Beijing, China, during September and October. These fruit cuttings were prepared by the authors with grape winter woods kindly provided by Mr. Kexu Cui (Shangri-La winery). Plants were grown in 1 L pots filled with a substrate mixture containing perlite, vermiculite and fine sand (v:v:v = 1:1:1). Plants were automatically irrigated 3 to 7 times per day with full-strength Hoagland’s solution. When the sixth leaf appeared, additional light treatment was conducted. LED lamps were used as supplemental light sources and were placed vertically above the plants. In the present study, two overhead light treatments were applied: natural light + white light (WW, as control) and natural light + far-red light (WFR, far-red LEDs peak at 730 nm), with three biological replicates for each treatment and each biological replicate with 9 cuttings. The light intensity of the supplemental white and far-red light was 101 µmol m^− 2^ s^− 1^ (Fig. 1), which was measured with a light spectrometer (LI-180, LI-COR, Nebraska, USA) at 40 cm below the lamps during the night without any natural light. To prevent light interference, white and far-red supplemental plants were separated by two layers of light-impermeable shade cloth (Fig. 1). The intensity and quality of the main light were measured with a light spectrometer (LI-180, LI-COR) at interval of 2 h from sunrise to sunset (Supplementary Fig. S1), and the main light intensity at canopy level was at about 700 µmol m^− 2^ s^− 1^ at noon. The proportion of additional white and far-red radiation to the total radiation was about 12.61% at noon.

The starting time of additional light treatment was synchronized with sunrise (6:00 am) and stopped at 10:00 pm. The photoperiod was 16 h/8 h, day/night. The temperature and relative humidity were synchronized with the daily changes in the glass greenhouse, and the maximum temperature was below 30℃ controlled by the temperature control system equipped in the greenhouse.

### Plant growth traits

The leaf area (LA) and stem length (SL) of all plants were nondestructively measured every three days from the first day after treatment (DAT = 1). Leaf area was calculated according to the equation of Junges [30]: LA (cm^2^) = (0.62*L*W-1.21)/100, where L and W represent the leaf length (mm) and maximum leaf width (mm), respectively. The leaf area of a whole plant was the sum of all leaf areas.

At the end of the one-month treatment, all experimental cuttings were destructively harvested. First, the leaves and stems from the sixth to the eighth leaf position, as well as the nonlignified fine roots, were collected and immediately frozen in liquid nitrogen and stored at -80 °C for RNA-seq and carbohydrate product analysis. Second, the remaining parts of the cuttings were separated into three compartments, leaves, stems and roots, and weighed for fresh weight. Finally, these three compartments were dried in the oven for 72 h at 80 °C to measure dry weight. Specific leaf weight (SLW, mg/cm^2^) was determined by the ratio of the single leaf dry weight and the corresponding single leaf area.

Sucrose, fructose, glucose and starch were extracted from 500 mg fresh powder. The powder was extracted with 2 ml deionized water, incubated at 80 °C for 15 min, and then centrifuged at 2400 g for 5 min. Supernatants were collected for sucrose, fructose and glucose analysis, and precipitates were collected for starch analysis. The contents of glucose, fructose and sucrose were measured enzymatically with an automated microplate reader (Synergy HIMF, BioTek, USA) using the D-Fructose/D-Glucose Assay Kit (K-FRUGL, Megazyme, Ireland) and sucrose invertase (E-INVPD, Megazyme, Ireland). For starch analysis, the precipitates were first decomposed into glucose by acid hydrolysis, and then the starch content was calculated by measuring the glucose content using an anthrone colorimetric method, according to the manufacturer’s instructions for the Starch Content Assay Kit (Cat^#^BC0700, Solarbio, China).

### SPAD measurements

In the present study, SPAD value was measured twice of each leaf to present the dynamic effects of leaf chlorophyll content during far-red supplemental treatment. Seven and 25 days after initiating supplemental light treatments, SPAD was measured for each leaf using a hand-held chlorophyll meter (SPAD-502, Konica-Minolta, Japan).

### Leaf net photosynthetic rate

In order to explore the effect of far-red light on grape plantlets, leaves of range 2 (formed before the treatment) and range 7 (emerged after treatment) were chosen for the leaf net photosynthetic rate measurement by a portable photosynthesis system (LI-6400XT, LI-COR, USA) equipped with a leaf cuvette chamber. The CO_2_ concentration and relative humidity within the leaf cuvette were similar to the plant growth environment inside the glass greenhouse, maintained at 500 ± 10 µmol mol^− 1^ and 50% ± 10%, respectively.

### Light response curves

The light response curve was measured within the leaf chamber built-in red/blue LED light of LI-6400XT. The fan was set to reach a flow rate to the sample cell at 500 µmol s^− 1^. The ratio of red and blue light in the leaf chamber was 9:1. Prior to measurement, the leaves were subjected to light induction with a light intensity of 2000 µmol m^− 2^ s^− 1^ for 30 min. After that, the light response curve was measured at the following light intensities: 2000, 1500, 1200, 1000, 750, 500, 250, 150, 100, 50, 20, and 0. Subsequently, measured data were fitted by a modified rectangular hyperbola formula [31], and the maximum net photosynthetic rate (Pn_max_) was obtained with the same method [31]. Meanwhile, parameters of stomatal conductance, transpiration rate (E) and water use efficiency were also recorded. Water use efficiency was calculated as Pn/E, which is extensively used in the literature [32].

### Transcriptome RNA sequencing and analysis

Leaves, stems and roots of grapevine plants grown under far-red light and white light supplementation conditions were harvested for RNA-seq analysis (Biomarker Technologies, Beijing, China). Total RNA was extracted using the plant total RNA isolation kit (Biomarker). The quality and quantity of RNA were analysed by a NanoDrop 2000 and an Agilent 2100 Bioanalyzer, respectively. Subsequently, a cDNA library was constructed using the VAHTS mRNA-seq V3 Library Prep Kit for Illumina (Vazyme, China) and sequenced with the Illumina NovaSeq 6000 sequencing platform. The paired-end reads were cleaned and trimmed with Trimmomatic version 0.39, and then high-quality reads were mapped to the v2.1 version of grape reference genome PN40024 12X by STAR (v.2.7.9a). The relative gene expression was calculated by RSEM (v1.3.1). The differentially expressed genes (DEGs) were selected with a threshold of an adjusted p value below 0.05 (*P* adj < 0.05) and |log2FC| > 1 by the DESeq2 R package (v1.34.0). Gene Ontology (GO) enrichment analysis and Kyoto Encyclopedia of Genes and Genomes (KEGG) analysis were carried out with AgriGO (v2.0) and ShinyGO (v0.75), respectively. Pearson’s correlations between DEGs and target traits were conducted with R software (v4.0.5), and networks were visualized with Cytoscape (v3.9.0).

### Statistical analysis and visualization

The experiments were conducted with three biological replicates and each biological replicate contained 9 cuttings. Significantly different difference analysis was performed with the Duncan’s multiple range test with a threshold of *P* < 0.05. The normality of the variable distribution was verified with Shapiro-Wilk test in R [33]. All data analysis in this study was conducted with R software (v4.0.5). Data were visualized with R and TBtools (v1.098691).

## Results

### Effect of far-red light on grape plantlet growth

**Fig. 2:**
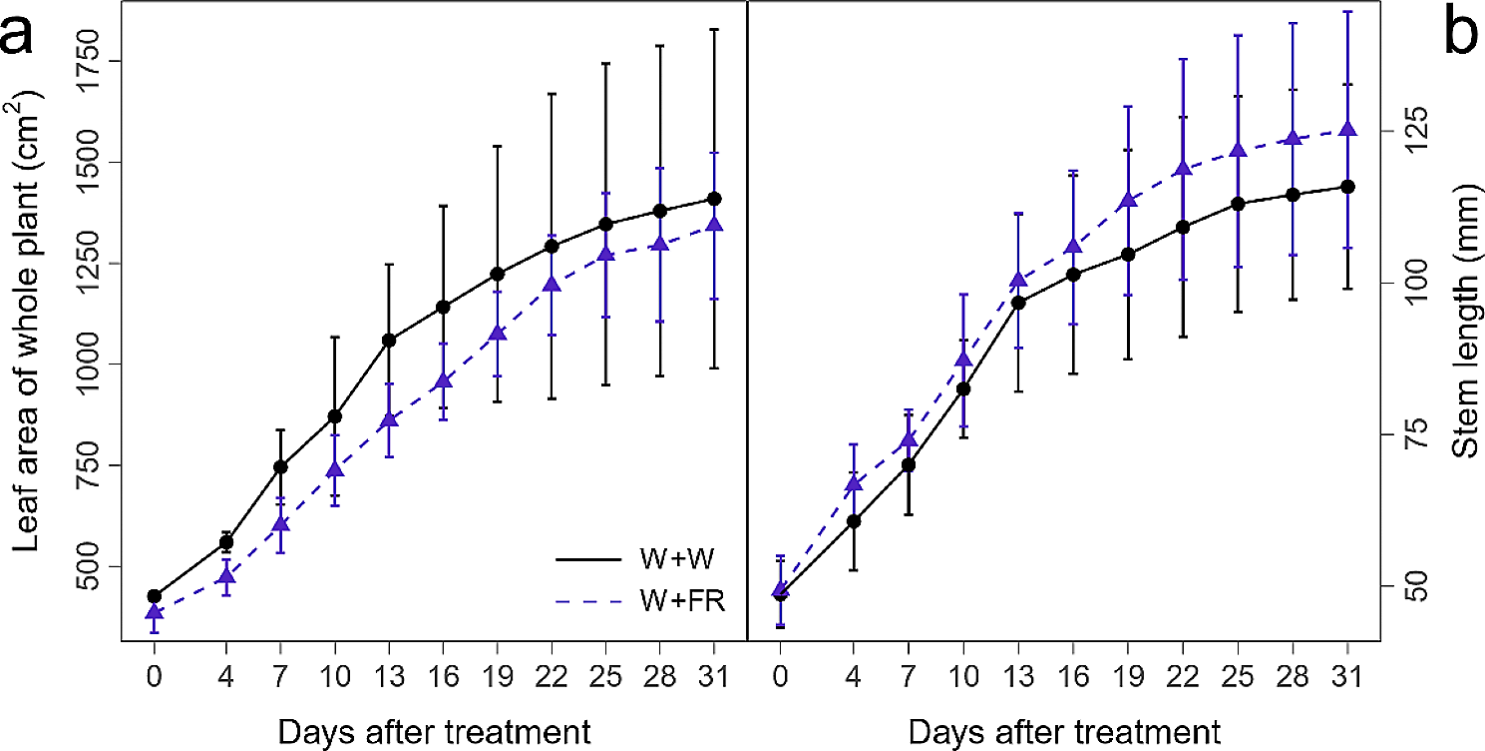
Effect of supplemental far-red light (W + FR) on the leaf area of the whole plant (**a**) and stem length (**b**) in comparison with vines grown under white light (W + W). Data are expressed as the means ± SEs (*n* = 3)

Compared to the control (WW), plant morphological traits were differentially affected by supplemental far-red light (WFR). The supplemental far-red light slightly decreased the leaf area of the whole plant while increased the stem length throughout the entire course of the treatment (Fig. 2), which was in line with the typical shade avoidance syndrome.

**Fig. 3:**
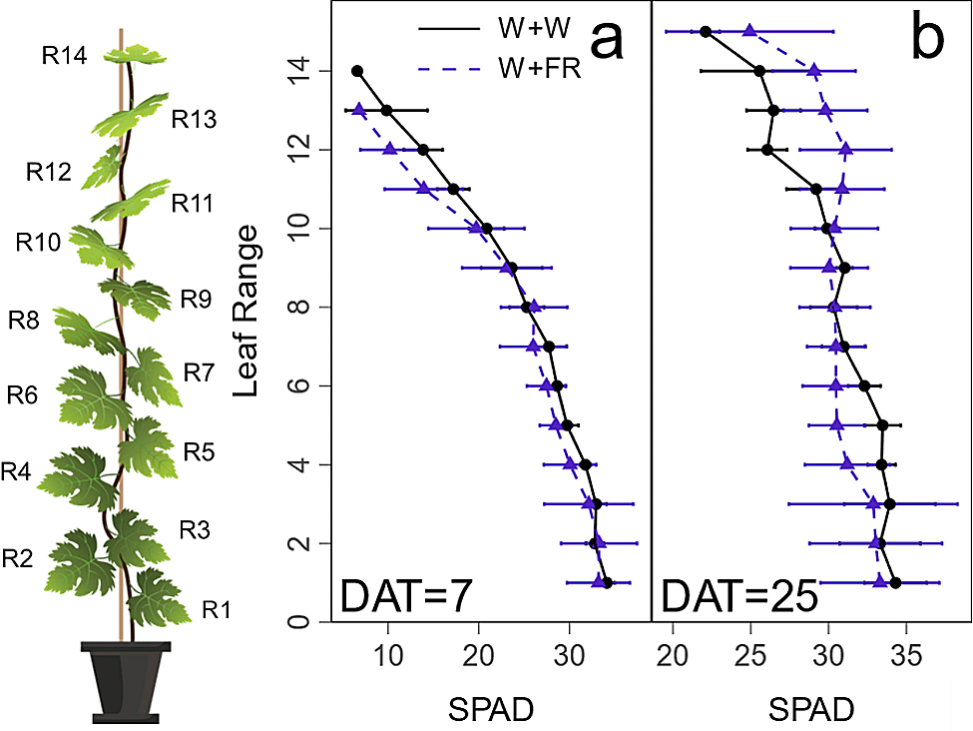
Effect of additional far-red light (W + FR) on physiological traits of grape plantlets in cooperation with white light (W + W). The chlorophyll content was measured at 7 days (**a**) and 25 days (**b**) after treatment. R1 to R14 indicate the blade positions within the canopy. Range 1 denotes the lowest leaf position. Data are expressed as the means ± SEs (*n* = 3)

Compared with WW, supplemental far-red light affected the spatiotemporal distribution of the leaf chlorophyll content (SPAD, Fig. 3). At the early stage, at 7 DAT, the SPAD values were nearly identical between WW and WFR regardless of the leaf position within the canopy (Fig. 3a). With the prolongation of the WFR treatment, at 25 DAT, far-red increased the SPAD value for higher range leaves (range 11–15) but decreased for leaves at lower range in comparison with those under WW (Fig. 3b). These data indicated that long-term far-red light supplementation affected the spatial distribution of chlorophyll content in leaves as a function of their positions within the canopy.

### Effect of far-red light on photosynthesis

**Fig. 4:**
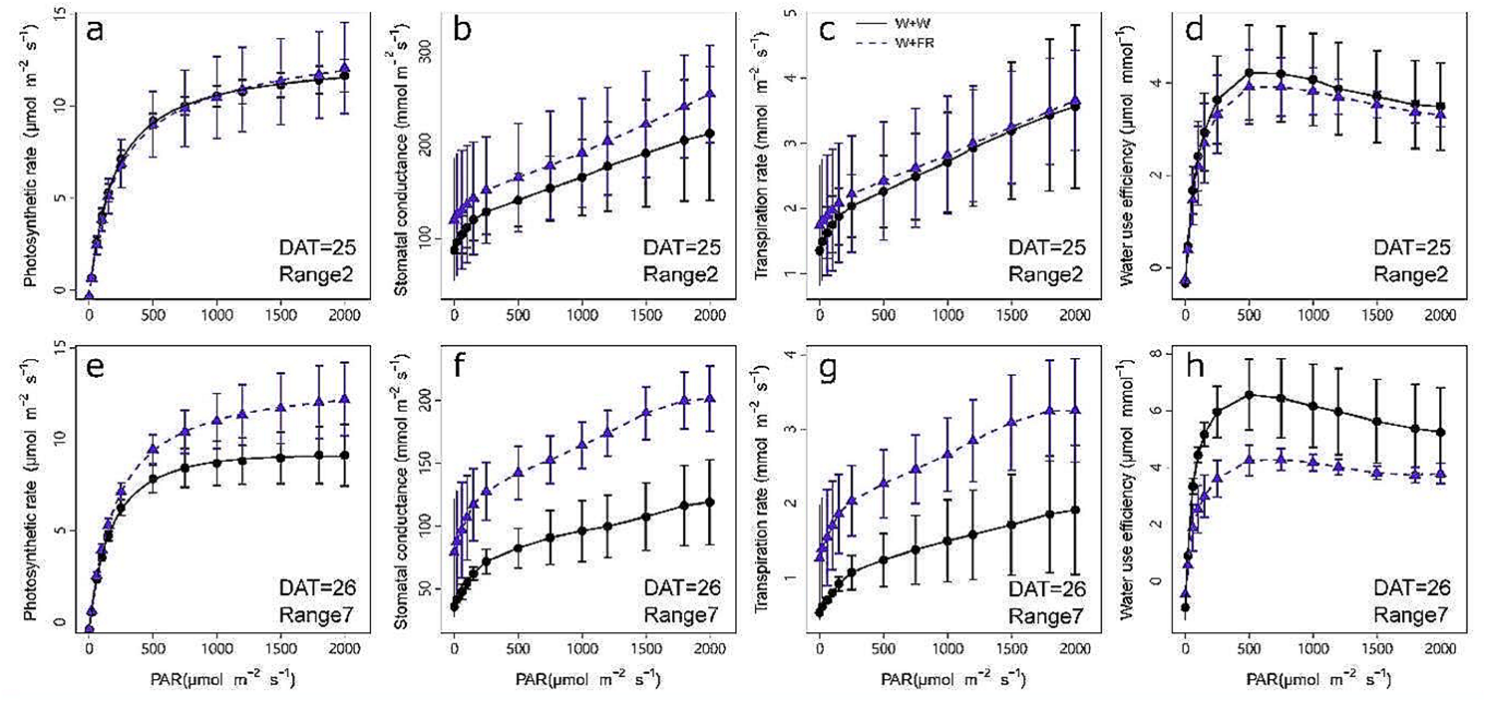
Effects of the additional far-red light (W + FR) on the light response curve of leaf gas exchange in cooperation with white light (W + W). Light response curve of photosynthesis rate (**a**, **e**), stomatal conductance (**b**, **f**), transpiration rate (**c**, **g**) and water use efficiency (**d**, **h**) of the range 2 leaf (**a**-**d**) and the range 7 leaf (**e**-**h**). Data are expressed as the means ± SEs (*n* = 3)

The light response curves of photosynthesis in leaves of range 2 and range 7 were measured at 25 and 26 DAT, respectively. The WFR enhanced the photosynthetic potential of the range 7 leaf but did not affect that of the range 2 leaf (Fig. 4). For the range 7 leaf, WFR greatly increased the net photosynthetic rate by 31.72% when PPFD > 150 µmol m^− 2^ s^− 1^ in comparison with their counterparts of the same light intensities under WW. On the other hand, no effect of WFR was observed when PPFD < 150 µmol m^− 2^ s^− 1^ (Fig. 4e). Meanwhile, the WFR significantly increased the stomatal conductance (Fig. 4f) and transpiration rate (Fig. 4g) but decreased the water use efficiency (Fig. 4h). The intensity of main light of range 7 leaf was higher than range 2 during the day from sunrise to sunset, while the quality of the main light was the same for the two ranges (Supplementary Fig. S1). In addition, both under WW and WFR condition, range 2 exhibited higher photosynthetic rate, stomatal conductance and transpiration rate, as well as lower water use efficiency than range 7 (Supplementary Fig. S2).

### Effect of far-red light on dry mass and carbohydrate accumulation

**Fig. 5:**
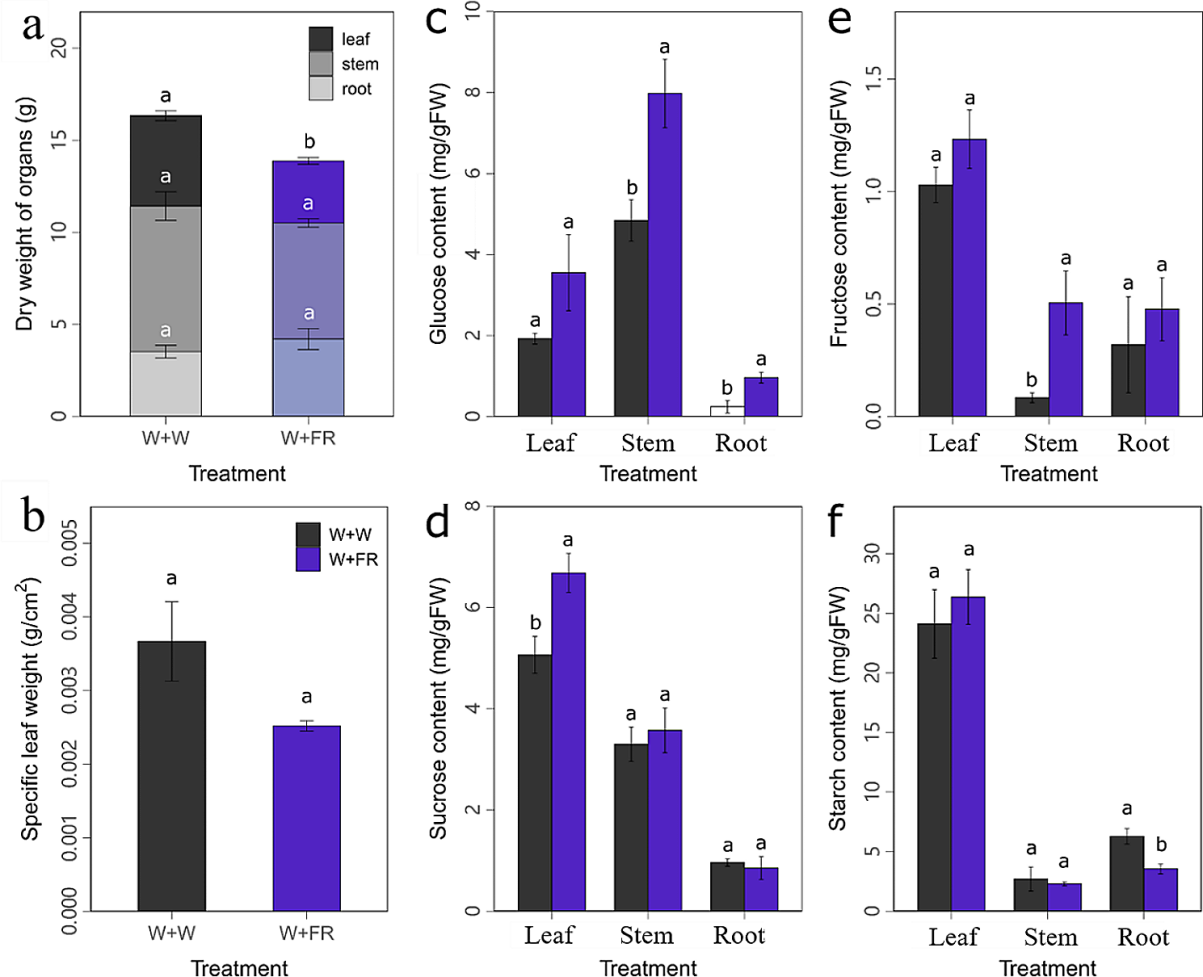
Effects of far-red light (W + FR) on dry mass and carbohydrate content in comparison with white light (W + W). Effect of the additional far-red light on the dry masses of different organs (**a**), specific leaf weight (**b**), contents of glucose (**c**), sucrose (**d**), fructose (**e**) and starch (**f**) in different organs. Data are expressed as the means ± SEs (*n* = 3). Different letters above the bar graphs represent significant differences (*P* ≤ 0.05)

The WFR decreased the whole plant dry mass mainly by reducing the leaf dry mass by 31.36% (Fig. 5a), as well as a slight decrease in the specific leaf weight (Fig. 5b), in comparison with WW. On the other hand, the dry masses of stems and roots were not significantly affected by the WFR (Fig. 5a).

The WFR significantly increased the content of soluble sugars in different organs but reduced starches in roots in comparison with WW (Fig. 5c-f). The contents of glucose in stems and roots were significantly increased by 64.70% and 2.93 times (Fig. 5c), respectively, and the content of fructose in stems was significantly increased by 5.01 times (Fig. 5e). The content of sucrose in leaves was significantly increased by 31.96% (Fig. 5d), while the starch content in roots was significantly decreased by 43.50% (Fig. 5f).

### Transcriptome remodelling in response to far-red light in different organs

**Fig. 6:**
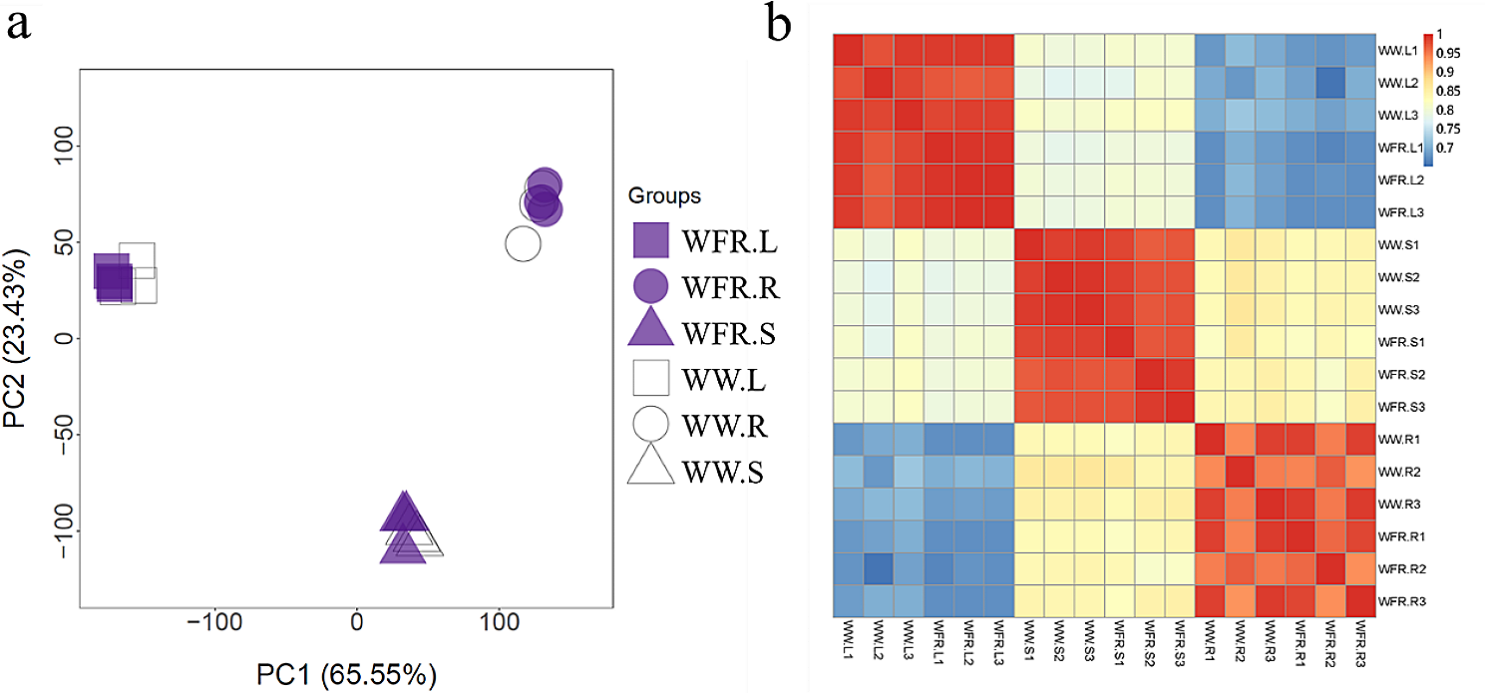
Organ specificity of transcriptomes under supplementation with far-red light. Principal component analysis (**a**) and correlation analysis (**b**) of the 18 transcriptome samples. In the legend in (**a**), WW and WFR indicate the white light control and far-red light supplementation treatment, respectively; the letter L, R and S denote leaves, stems and roots, respectively. In the axis labels of (**b**), the numbers 1, 2 and 3 denote the sample ID of biological replicates

To decipher the mechanisms underlying the effect of additional far-red light on the physiological and biochemical variations, a transcriptomic analysis was performed using the leaves, stems and roots harvested at 26 DAT. This produced a total of 18 samples with 2 treatments (WW vs. WFR), 3 organs (leaf, stem and root), and 3 biological replicates. A principal component analysis (PCA) showed that these samples were mainly discriminated by organs, with the three biological replicates tightly grouped (Fig. 6a). For the same organ, there were clear separations between WW and WFR, indicating that the WFR indeed affected organ transcriptomes. Similarly, correlation analysis revealed that there was a high correlation within each group, supporting the reliability of three biological replicates (Fig. 6b). In addition, leaves and stems and stems and roots showed high correlations, respectively, but there was a low correlation between leaves and roots. These results assured that the transcriptome data were reliable and could be used for further analysis.

**Fig. 7:**
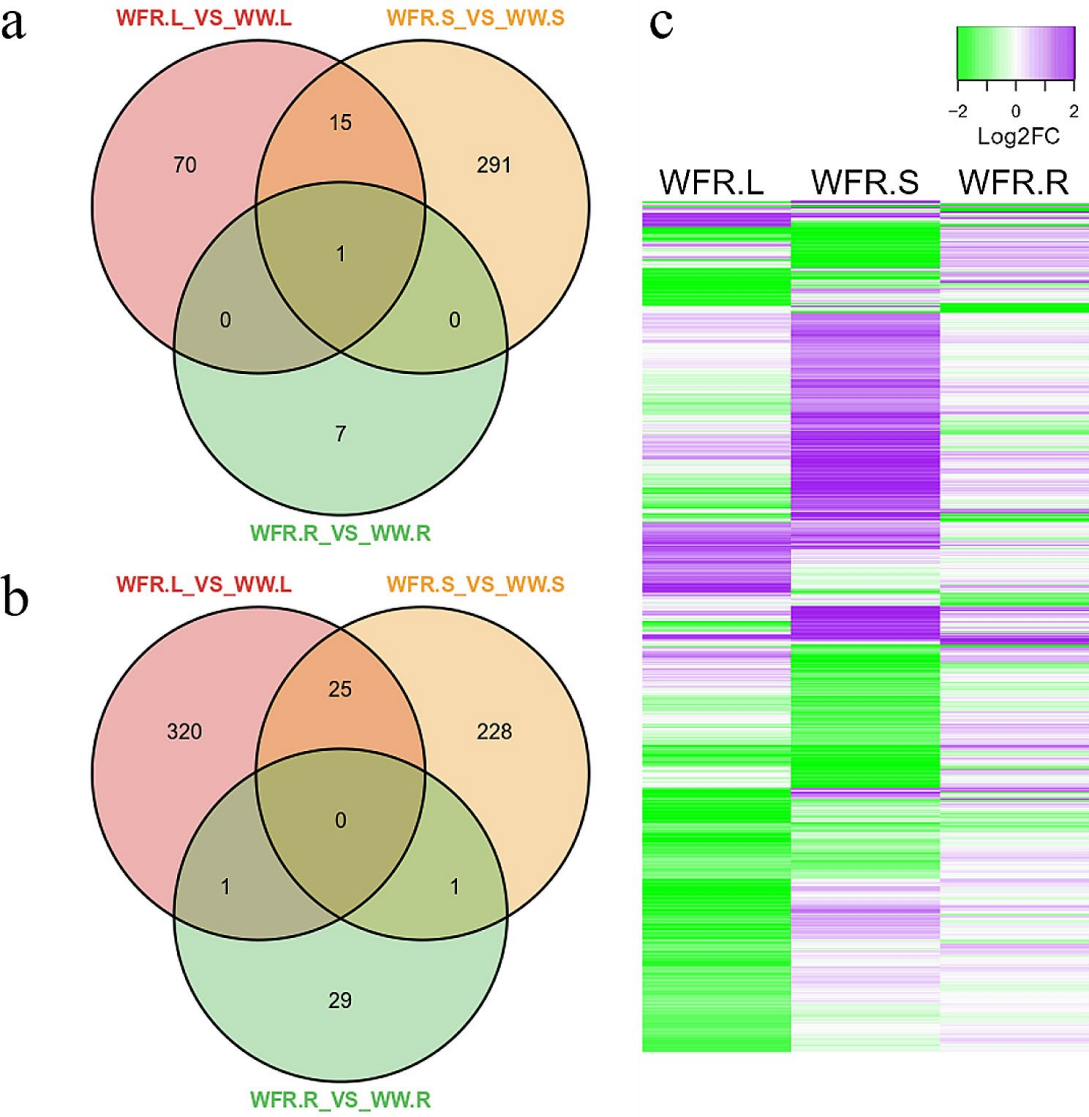
Venn diagram and clustering analysis of differentially expressed genes in different organs under the effects of additional far-red light (WFR) in comparison with white light (WW). Venn diagram of upregulated (**a**) and downregulated DEGs (**b**) and expression clustering analysis of DEGs (**c**). The color scale in (**c**) indicates the differential expression fold change, in which purple represents upregulation and green represents downregulation of gene expression. In the legend, WW and WFR indicate the white light control and far-red light supplementation treatment, respectively; the letter L, R and S denote leaves, stems and roots, respectively

Differentially expressed genes (DEGs) were identified by comparing the WFR with WW for a given organ. This provided three comparisons, including WFR. L vs. WW. L, WFR. S vs. WW. S and WFR. R vs. WW. R, with R, S and L for roots, stems and leaves, respectively. A total of 982 unique DEGs were identified in the three comparisons (Fig. 7, Supplementary Data Set S1), including 384 upregulated (Fig. 7a) and 604 downregulated (Fig. 7b) DEGs, and 6 common DEGs were identified between upregulated and downregulated genes, as these 6 genes were differentially regulated by WFR between organs. Notably, the WFR induced more downregulated DEGs than upregulated DEGs for a given organ. Among the three organs, the WFR induced the highest numbers of DEGs in stems, with 561 DEGs, compared to 432 and 39 DEGs in leaves and roots, respectively (Fig. 7a and b). The number of DEGs identified in different organs suggested that the aboveground organs (leaves and stems) were more responsive to far-red light than the belowground organs (roots) and that the far-red light responses are more local than systematic.

To decipher the overall response trends of these 982 DEGs in the three organs, clustering analysis was conducted (Fig. 7c). The results showed that most of the DEGs responded differentially between organs. For example, the upregulated genes in leaves could exhibit downregulation, upregulation or no significant changes in stems and roots. This confirmed the results of the Venn diagram showing very few common genes between different comparisons (Fig. 7a and b). Only one common gene that was upregulated by supplementation with far-red light in these three organs, VIT_208s0040g00820, encoding a cytochrome P450 protein CYP94D25 [34] or CYP94D1 [35], was upregulated by 2.99-, 3.62- and 3.47-fold in leaves, stems and roots, respectively. Previous studies have demonstrated that genes of the CYP94 family mediate jasmonic acid homeostasis [36], but the function of this common gene of the CYP94D subclade is still unknown [37].

### GO analysis

**Fig. 8:**
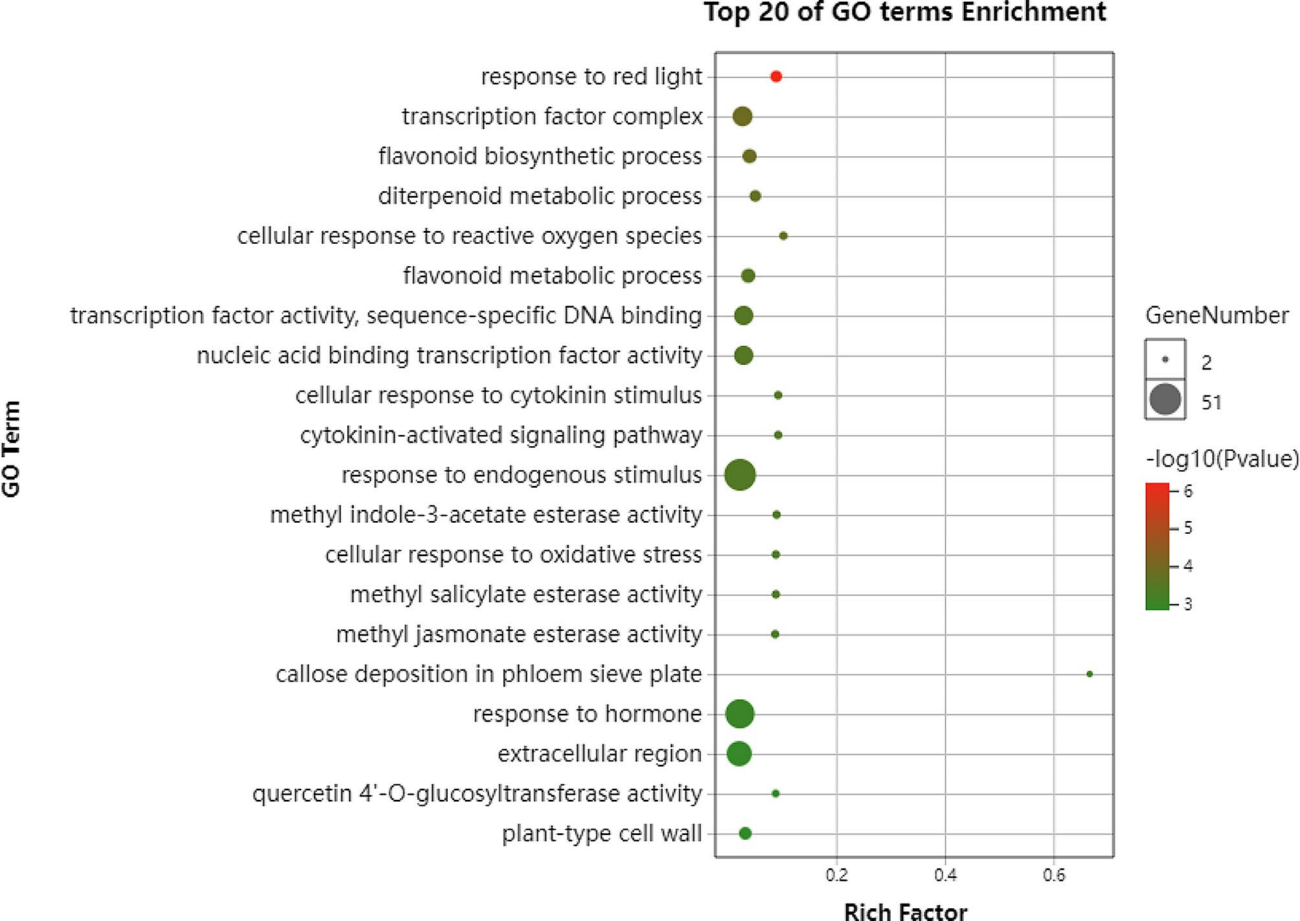
GO functional enrichment of DEGs in leaves

**Fig. 9:**
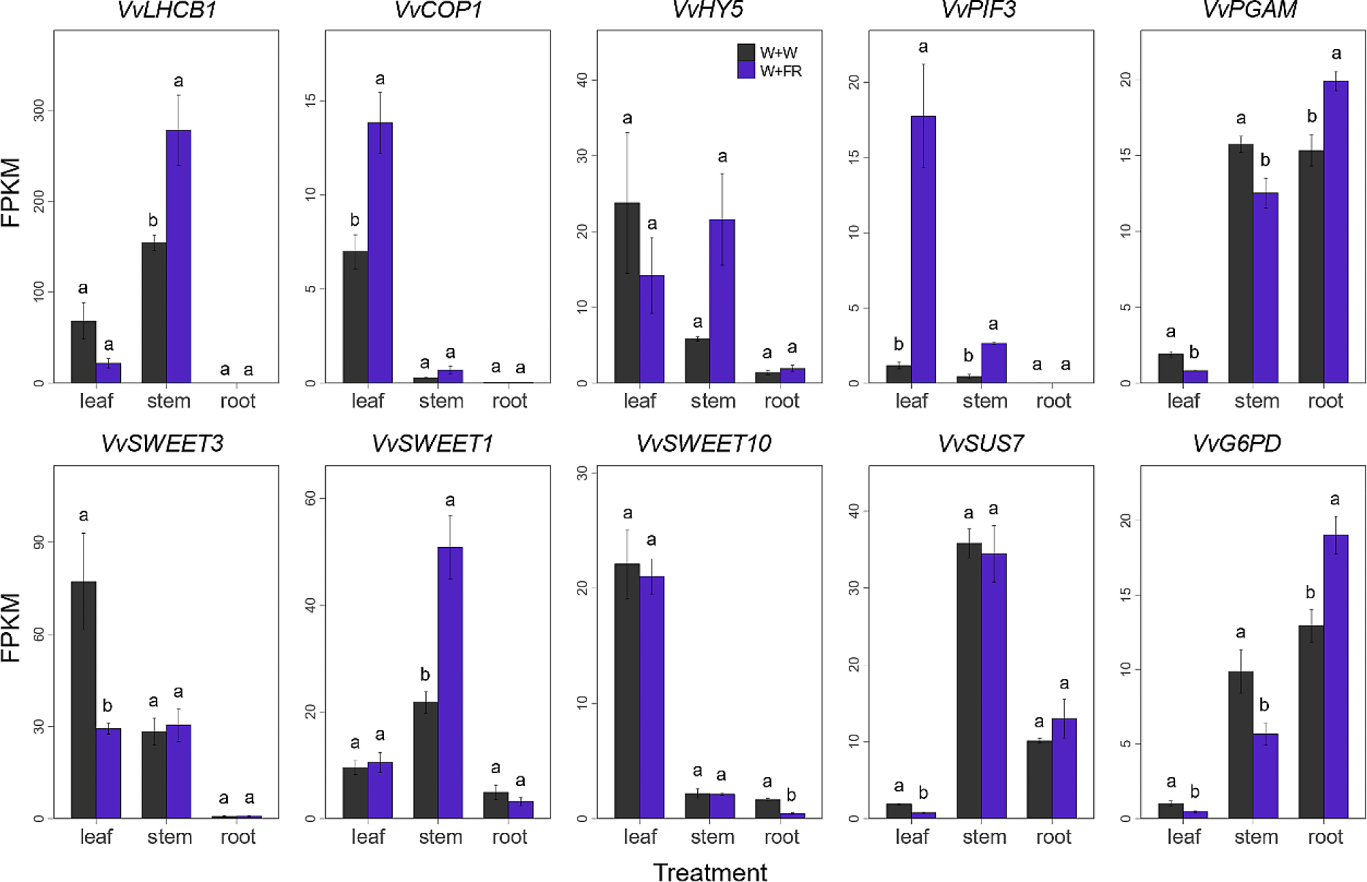
Effects of far-red light on the expression of genes related to photosynthesis, light signal transduction and carbon metabolism. Error bars are expressed as the SE of triplicates. Different letters above the bar graphs represent significant differences (*P* ≤ 0.05)

To gain insight into the functional categories impacted by the WFR, GO enrichment analysis was conducted with DEGs identified in leaves, stems and roots. These DEGs were enriched in a wide range of physiological processes, including light response, hormone signaling, secondary metabolism, metal ion transport and starvation response, indicating that far-red light deeply altered a series of plant growth processes (Fig. 8 and Supplementary Fig. S3). Similarly, transcription factor complex, flavonoid biosynthetic process, diterpenoid metabolic process, hormone signaling and iron ion binding were common terms enriched in the three organs (Fig. 8 and Supplementary Fig. S3). Strikingly, the light responsive category was found only in leaves (Fig. 8). Response to red light was the most significantly enriched GO term in leaves, with 12 genes (Supplementary data Set S2). Among them, *VIT_207s0005g02220* encodes the critical light-harvesting complex II chlorophyll a/b binding protein VvLHCB1 (Fig. 9 and Supplementary Data Set S2), *VIT_217s0000g06360* and *VIT_217s0053g00990* encode alpha-expansin, which is related to cell expansion, and *VIT_218s0001g05690* encodes a phosphatase, and its Arabidopsis homolog PAPP2C is a phytochrome-associated protein that interacts in the nucleus with phyA and phyB to regulate the red light signaling pathway. In addition, 5 MYB-related transcription factors related to responsive to light stimulus, one bHLH transcription factor, and WD repeat-containing protein RUP2-like were also identified in this term.

### Expression of genes related to photosynthesis, light signaling, carbon metabolism and sugar transport

The top 50 up-regulated DEGs in leaf enriched to GO terms of ‘response to radiation’, ‘response to light stimulus’ in leaves, while these GO terms were not enriched in the 50 top up-regulated DEGs in stems or roots (Supplementary Data Set S1 and Supplementary Fig. S4). Considering the most evident phenotypic responses to the far-red supplementation identified in the current study were related to photosynthesis and carbohydrate accumulation, we focused our analysis on the genes involved in these processes. To this end, known genes related to these pathways were explored in more detail. Nine DEGs were identified, including a light-harvesting antenna protein of PSII system encoding gene (*VvLHCB1*), 2 genes coding for light signaling transduction (*VvCOP1* and *VvPIF3*), 3 genes coding for carbon metabolite (*VvG6PD*, *VvSUS7* and *VvPGAM*) and 3 sugar transporters encoding genes (*VvSWEET1*, *VvSWEET3* and *VvSWEET10*). The well-known light signaling player *VvHY5* was not included in the DEGs, but its expression was also illustrated (Fig. 9).

Expression analysis of these 10 genes revealed that they responded differentially to the WFR among organs (Fig. 9). Except for three carbon metabolism genes, the other 7 genes were prominently expressed in leaves and stems, while in roots, their expression was extremely low or hardly detected. The WFR had reverse effects on *VvLHCB1* transcription between leaves and stems, which was slightly decreased in leaves but significantly increased in stems. In contrast, both *VvCOP1* and *VvPIF3* were upregulated in leaves and stems by WFR.

In line with the sugar concentrations, the transcript abundance of sugar transporters, including *VvSWEET1*, *VvSWEET3* and *VvSWEET10*, was altered by the WFR treatment. The glucose transporter *VvSWEET1* was greatly increased in stems under WFR treatment, which was consistent with the increased content of glucose in stems (Fig. 5c). *VvSWEET3* was significantly decreased in leaves after WFR treatment, which coincided with the high content of sucrose in leaves (Fig. 5d), suggesting that *VvSWEET3* may be a critical sucrose transporter. *VvSWEET10* belongs to clade III SWEET, which mediates sucrose efflux in Arabidopsis [38, 39]. Herein, its expression was significantly downregulated in roots and not affected in leaves and stems under WFR conditions, which was in agreement with the decrease in sucrose accumulation in roots (Fig. 5d). Notably, the three genes related to carbon metabolism, *VvG6PD*, *VvSUS7* and *VvPGAM*, shared the same transcript profile that was downregulated in leaves and stems but increased in roots by WFR. Moreover, their transcript abundances in leaves were consistently lower than those in stems and roots, regardless of light treatments, which was consistent with the significant decrease in dry weight in leaves and slight decrease in stems but slight increase in roots (Fig. 5a). In addition, VvG6PD was also an important enzyme in metabolite flow from glycolysis to pentose phosphate pathway involved in nucleotide biosynthesis and redox balance. So key genes related to the maintenance of the redox status [40] were analysed in the present data, and results showed that the expression of *VvSOD*, *VvAPX* and *VvCAT* were not significantly affected by WFR (Supplementary Fig. S5). This suggests that the far-red light supplementation may not affect the redox status of grapevine.

### Correlation network analysis

**Fig. 10:**
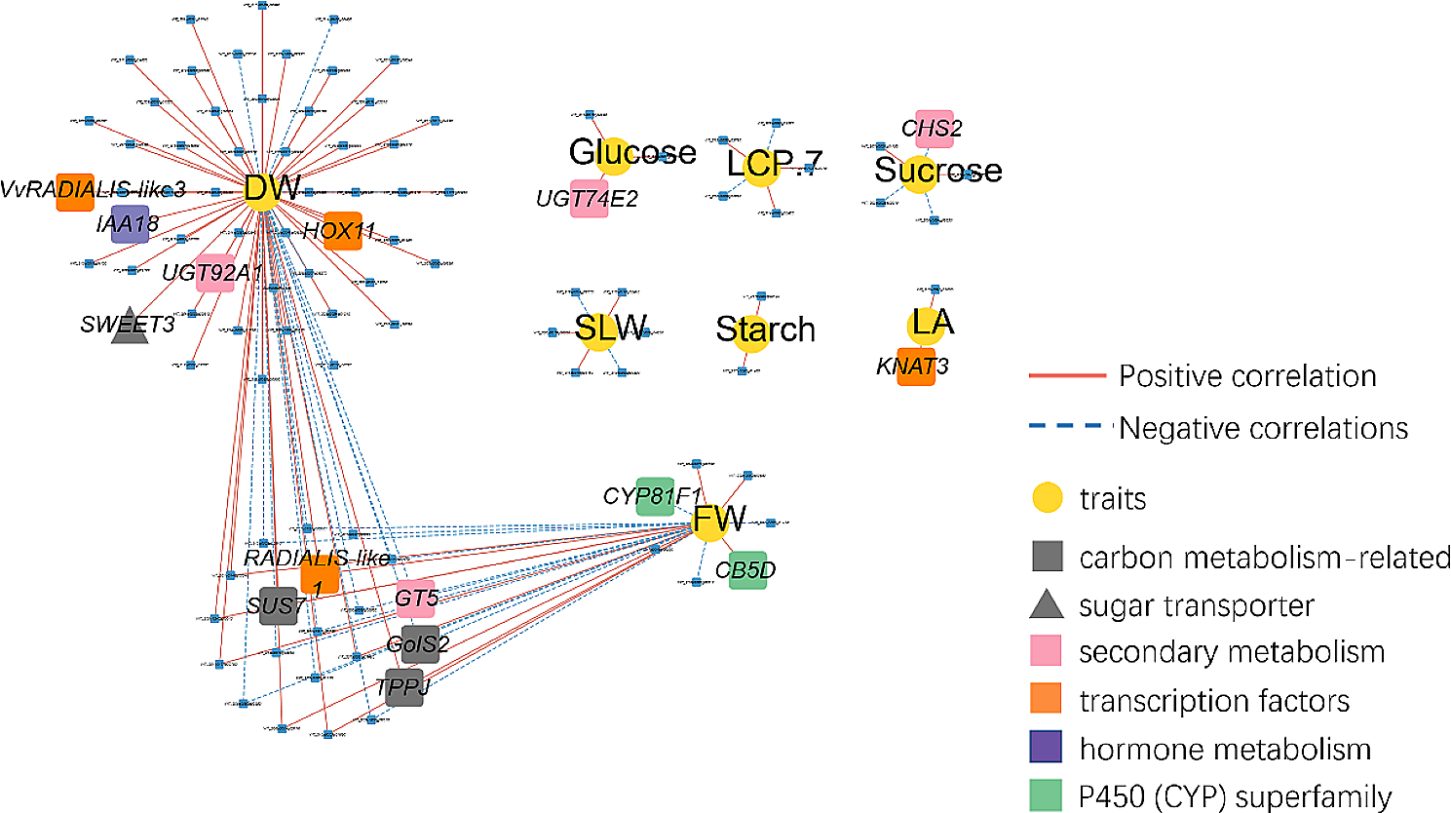
Correlation network between traits and DEGs in leaves. *VvSUS7*, sucrose synthase 7; *VvGolS2*, galactinol synthase 2; *VvTPPJ*, trehalose-phosphate phosphatase J; *VvSWEET3*, Sugars Will Eventually be Exported Transporter 3; *VvGT5*, 3-*O*-glucosyltransferese 5; *VvUGT74E2*, UDP-glycosyltransferase 74E2; *VvCHS2*, chalcone synthase 2; *VvUGT92A1*, UDP-glycosyltransferase 92A1; *VvRADIALIS-like3*, RADIALIS-LIKE SANT/MYB 3; *VvRADIALIS-like1*, RADIALIS-LIKE SANT/MYB 1; *VvHOX11*, homeobox-leucine zipper protein HOX11; *VvKNAT3*, homeobox protein knotted-1-like 3; *VvIAA18*, auxin-responsive protein IAA18; *VvCB5D*, Cytochrome b5 heme-binding domain-containing protein; *VvCYP81F1*, isoflavone 3’-hydroxylase

Correlation network analysis was conducted to investigate the interrelationship between all the measured physiological traits and DEGs and to further identify novel candidate genes. Only those correlations with an absolute correlation coefficient > 0.80 and an adjusted *P* value < 0.05 were selected. In leaves, a total of 124 pairs of significant trait-gene correlations (between 103 genes and 8 traits) were screened, including 83 positive and 41 negative correlations (Fig. 10). The network can be divided into 3 connected modules and 6 isolated modules. The first module represented genes highly connected to leaf dry weight, including sugar transporters, secondary metabolism-related genes, genes encoding hormone-responsive proteins and transcription factors. The second module was related to leaf flesh weight, and two cytochrome P450-encoding genes were found. The third module showed common links to leaf dry weight and leaf flesh weight, including carbon metabolism genes (*VvSUS7*, *VvGolS2* and *VvTPPJ*), a secondary metabolism gene (*VvGT5*), and a transcription factor related to light responsiveness (*VvRADIALIS-like1*) (Supplementary Fig. S6 and Supplementary data Set S3). The 6 isolated modules were arranged for 6 specific physiological traits, including the concentrations of leaf glucose, sucrose and starch, as well as the SLW, LA, and LCP.7. Similarly, a correlation network between 104 genes and 6 traits was constructed in stems (Supplementary Fig. S7). Evidently, the content of fructose in stems was found to be significantly correlated with 88 genes, including carbon metabolism, sugar transporter, photoreceptor protein, photosynthesis, light responsive protein, secondary metabolism, transcription factors, hormone metabolism potassium transporter and ion channel protein.

## Discussion

### Far-red light altered the morphological traits of grape plantlets

Plants often alter their morphological structure to increase light absorption when exposed to shade or low F:FR conditions. The most prominent change is stem elongation [10]. Promotion of stem length by low R:FR is a common response to far-red light and is widespread among a wide range of species [41]. In tomato, internode length increased with increasing intensity of far-red light [42]. It was believed that stem elongation contributes to enhancing the light-foraging capacity in dense stands and enables plants to overtop competing vegetation [41]. Consistent with previous studies, the stem length was increased by the WFR in this study. Unlike the consistent responses of plant stems to far-red light, the effect of far-red light on leaf area varies with plant species and growth stages, ranging from inhibition to promotion [41, 42]. For example, far-red light increased leaf area in lettuce [1, 19, 23, 43, 44], geranium and snapdragon [21] and tomato [45, 46], *Dendrobium officinale* [46], Chinese Kale [47]. In other studies, the leaf area of tomato varied differently between growth periods, decreasing in the early stage and increasing in later stages under far-red light [42]. In the present study, far-red supplementation decreased the leaf area, which was in agreement with previous reports [48]. The decrease in leaf area may cause two reverse consequences for whole-canopy photosynthesis: the reduced leaf area will decrease the light interception area and may reduce photosynthesis, while the decreased leaf area may also reduce mutual shading between leaves within the canopy and increase the light interception of each single leaf [49]. The overall performance of whole-canopy carbon assimilation will be a result of counterbalancing the two aforementioned processes [5, 23]. In this study, the reduced leaf area led to a decrease in whole-plant dry mass, suggesting that whole-canopy carbon assimilation was reduced and that the first process dominated the far-red effect. Overall, grape plants progressively adjusted their morphological characteristics to adapt to novel light environments during long-term far-red light supplementation.

### Far-red light enhanced the potential photosynthesis efficiency

In this study, leaf chlorophyll presented distinct spatial-temporal distributions after a long WFR (Fig. 3). At 7 DAT, the early stage after far-red supplemental treatment, no difference was observed between the far-red light treatment and control condition. After long-term far-red treatment (25 DAT), the chlorophyll content increased in the leaves in the high range but decreased in the low range compared to the control. Previous studies showed that far-red irradiation reduced the leaf chlorophyll content in lettuce and kale [19, 50], which was inconsistent with our results for high-range leaves. This discrepancy might be caused by the sampling strategy, where ignoring the spatial variation in leaf chlorophyll content may mask the fine effect of far-red light.

Since the chlorophyll varied among leaves at different layers, the photosynthesis efficiency quantified from different leaves was also different, which was increased in upper layer leaves but was not affected in lower layer leaves (Fig. 4). All of these parameters related to photosynthesis efficiency, such as photosynthetic rate, stomatal conductance transpiration rate and water use efficiency, were consistent between the far-red light and white light conditions when quantified at range 2 leaf. These results indicated that far-red light supplementation did not impact the photosynthesis of lower layer leaves. In contrast, for the upper leaf at range 7, the photosynthetic rate, stomatal conductance and transpiration rate increased markedly under far-red light supplementation, and the Pn max significantly increased by 31.72% when compared to white light conditions, suggesting that far-red light supplementation effectively elevated leaf potential photosynthesis, which was consistent with previous studies [24].Moreover, this result was somewhat expected, because the leaves of range 2 were formed before the treatment, while the range 7 leaf was emerged after treatment and therefore was most likely more evidently influenced by the light quality. Besides, for the same cutting, even though the intensity of main light of range 7 was higher than range 2 (Supplementary Fig. S1), the maximum photosynthetic rate of range 2 leaf was still higher than range 7 both under WW and WFR condition (Supplementary Fig. S2), indicating that leaf age may play an important role in affecting photosynthesis, as discovered in previous study in grapevine [51].

### Far-red light influenced photoassimilate accumulation and allocation

Sucrose is the main form of transportation of photoassimilates in grapevine [52], starch is the storage form of photoassimilates for most plants [53], and glucose and fructose are the main forms of soluble sugars [52]. Herein, in comparison with white light supplementation, far-red light increased the content of photoassimilates (glucose, fructose, sucrose and starch) in leaves and stems, although to different extents, while it decreased the content of sucrose and starch in roots (Fig. 5). These results were consistent with previous studies in soybean and tomato showing that far-red light increased sucrose and starch contents in leaves [54, 55]. In contrast, strawberry leaves showed higher levels of sucrose and lower levels of starch in response to far-red light [56]. Taken together, it seems that the effect of far-red light on photoassimilate accumulation was species dependent. Nevertheless, far-red light indeed plays an important role in photoassimilate accumulation in many species, regardless of positive or negative effects. Besides, far-red supplementation had an effect on carbohydrate mobilization in roots compatible with starch concentration reduction and glucose increase.

Moreover, photoassimilate allocation among organs is also regulated by far-red light, often increasing partitioning to shoots rather than roots [15]. It was interesting to note that in tomato, additional far-red light increased the fraction of dry mass partitioned to fruit at a cost of reducing the partitioning proportion to leaves, which mainly resulted from increasing fruit sink strength by stimulating the expression of genes related to sugar transportation and metabolism under far-red light [13, 14]. In the present study, inconsistent with previous studies, far-red light significantly decreased the dry weight of leaves but did not affect the dry mass of stems and roots.

Subsequently, three genes related to carbon metabolism, *VvG6PD*, *VvSUS7* and *VvPGAM*, were identified with altered expression after far-red light supplementation. VvG6PD encodes a glucose-6-phosphate dehydrogenase, and its transcript level was highest in roots, followed by stems and leaves (Fig. 9). This gene was found to be differentially regulated among organs exposed to far-red light, which was decreased in leaves and stems but increased in roots (Fig. 9). Therefore, it was consistent with the increased content of glucose in the leaves and stems (Fig. 5). Interestingly, the homolog gene of *VvG6PD* in Arabidopsis *AtG6PD2* was found to be predominantly expressed in roots and induced by nitrate [57, 58]. Moreover, far-red light was able to induce shoot-to-root communication to coordinate carbon and nitrogen acquisition in the roots of Arabidopsis [59, 60]. These results indicated that *VvG6PD* may contribute to mediating glucose metabolism in aboveground organs (leaves and stems) and regulating the carbon-nitrogen balance in belowground organs (roots) in response to far-red light. In addition, the expression variation of *VvSUS7*, encoding sucrose synthase, was negatively correlated with the content of sucrose in the three investigated organs (Figs. 5 and 9), indicating that the altered sucrose content in response to far-red light may be attributed to the modified expression of *VvSUS7*, especially in leaves. *VvPGAM* encodes a phosphoglycerate mutase, which plays a key role in starch granule synthesis, and its homology is also highly induced by nitrate in Arabidopsis [61, 62]. In this study, *VvPGAM* was downregulated in the leaves and stems but upregulated in the roots (Fig. 9). However, its expression was not in line with the variation in starch in these organs (Fig. 5), suggesting that *VvPGAM* may also be involved in carbon-nitrogen balance during shoot-to-root communication under far-red irradiation. In addition, sugar transporters such as *VvSWEET1*, *VvSWEET3* and *VvSWEET10* were altered in response to WFR (Fig. 9). Except for *VvSWEET10*, the transcript abundances of *VvSWEET1* and *VvSWEET3* were well correlated with variations in glucose and sucrose in stems and leaves (Fig. 5), respectively, indicating that they may play a role in sugar transport in response to far-red light irradiation [13, 39].

### Identification of key genes in response to far-red light irradiation

More DEGs were identified in the aboveground organs (leaves and stems) than in the belowground organs (roots), suggesting that aboveground organs are more sensitive to far-red light radiation (Fig. 7). GO analysis revealed that the red light responsive term was significantly enriched in leaves (Fig. 8), suggesting that leaves are more sensitive to perceive far-red fluctuation in their ambient surroundings. In total, 9 DEGs involved in photosynthesis, light signal transduction, carbon metabolism and sugar transport were identified with altered expression in response to far-red radiation (Fig. 9). Among them, *VvLHCB1*, encoding a light-harvesting chlorophyll a/b binding antenna complex protein, was greatly induced in stems under far-red light conditions. In Arabidopsis, *LHCB1* was induced in low-light conditions [29] and bound with chlorophyll to form a complex that was involved in the harvesting and transporting of solar energy during photosynthesis. More importantly, the phosphorylation/dephosphorylation of the LHCB1 protein allows it to shuttle between PSI and PSII to restore the excitation balance of the two photosystems, thus playing an important role in state transitions [29]. In addition, *LHCB* also functions in multiple processes that are critical to plant growth, development, and abiotic stress response [63, 64]. For example, overexpression of *LHCB2* could improve shoot and root elongation in tobacco [64], which was consistent with the stem elongation observed in the current study. Taken together, these data indicated that *VvLHCB1* may have an important role in sensing and responding to far-red light supplementation in grapevine.

COP1 is the central repressor of plant photomorphogenesis [65]. It has been reported that low F:FR induces COP1 translocation into the nucleus, where it directly participates in the degradation of the positive regulator (HY5) by ubiquitination [66, 67] and promotes the accumulation of the negative regulator (PIF3) [67, 68], together contributing to the maintenance of skotomorphogenesis [69]. In the present study, *VvCOP1* was significantly upregulated in leaves, suggesting that far-red promotes its gene expression, which is in agreement with a previous study in *Brassica napus* [70]. PIF3 is also the key repressor of photomorphogenesis, and the accumulation of PIF3 in the dark requires the presence of COP1 [68]. Here, far-red light induced an increase in the expression of *VvPIF3* in leaves and stems, which was consistent with stem elongation, as identified in other study [70]. Taken together, *VvCOP1* and *VvPIF3* may play essential roles in grape plantlet responses to far-red light.

Additionally, from correlation analysis, two MYB-related transcription factors were identified, *VvRADIALIS-like1* and *VvRADIALIS-like3*, which were associated with fresh weight and dry weight traits. As described above, the dry mass of leaves was significantly decreased (Fig. 5), which was linearly correlated with the remarkably declined expression of *VvRADIALIS-like1* and *VvRADIALIS-like3* under far-red light supplement conditions (Supplementary data Set S3). In Arabidopsis, their homologous gene *RADIALIS-LIKE SANT/MYB 1 (RSM1)* positively regulates early photomorphogenesis under red light conditions [71]. Seedlings overexpressing *RSM1* displayed hooklessness and loss of gravitropism and showed short hypocotyls under red light [71]. Furthermore, RSM1 could directly interact with HY5, the key positive regulator of the plant light signaling pathway, to modulate seed germination and seedling development [72]. Collectively, these data indicated that *VvRADIALIS-like1* and *VvRADIALIS-like3* may have important roles in the regulation of the carbon metabolism pathway in grape leaves under far-red light supplementation conditions.

## Conclusions

In conclusion, additional far-red radiation elevated the maximum net photosynthetic rate, and subsequently increased the concentrations of glucose, fructose, sucrose and starch in leaf and stem to different extents. In root, the concentrations of glucose and fructose were increased while sucrose and starch were decreased. Consistently, the expression of genes related to photosynthesis and carbon metabolism was well correlated with these variations. Genes encoding light-trapping antenna *VvLHCB1*, light signaling pathway genes *VvCOP1* and *VvPIF3*, transcription factors *VvRADIALIS-like1* and *VvRADIALIS-like3*, genes encoding sugar transporter proteins *VvSWEET1* and *VvSWEET3* and carbon metabolic pathway genes *VvG6PD*, *VvSUS7* and *VvPGAM* were identified as candidate genes for further study of the regulation of photosynthesis and carbon metabolism by far-red light. It should be noted that the current work was conducted with young grapevine cuttings without fruits, which may have a different source-sink relationship than those of more developed plants. Further investigations need to be conducted to confirm the current conclusions with well-developed and fruited vines.

### Electronic supplementary material

Below is the link to the electronic supplementary material.

Supplementary Material 1

Supplementary Material 2

Supplementary Material 3

Supplementary Material 4

## Data Availability

The transcriptome data generated in the current study are available in the National Genomics Data Center under accession number CRA011017. All data used in this study are included in the manuscript and supplementary files.

## Abbreviations

PSI: Photosystem I
PSII: Photosystem II
WW: Natural light + white light
WFR: Natural light + far-red light
PAR: Photosynthetically Active Radiation
LA: Leaf area
SL: Stem length
DAT: Day after treatment
SLW: Specific Leaf Weight
PCA: Principal component analysis
DEG: Differentially expressed gene
