# Supplementary material for: Far-red light modulates grapevine growth by increasing leaf photosynthesis efficiency and triggering organ-specific transcriptome remodelling: Author
Source: BMC Plant Biol. 2024 Mar 15;24:189. doi: 10.1186/s12870-024-04870-7 (PMC10941557; doi:10.1186/s12870-024-04870-7)

Supplementary Fig. S1: The intensity (a) and quality (b) of the main light without far-red supplementation.


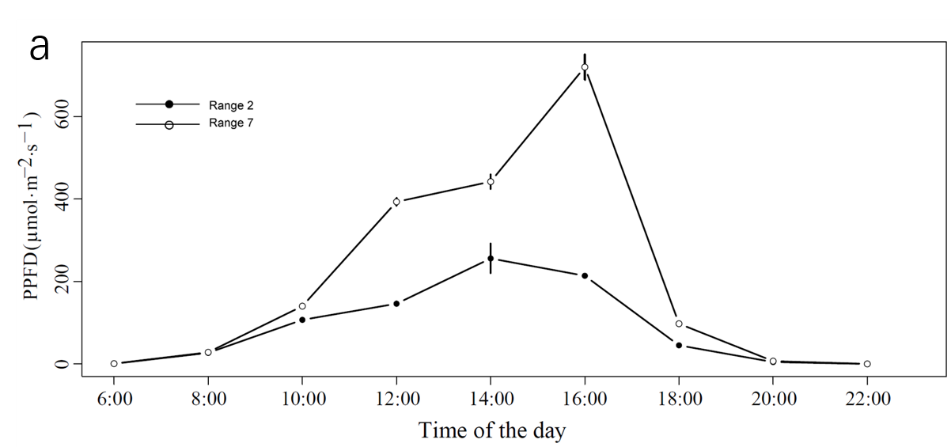


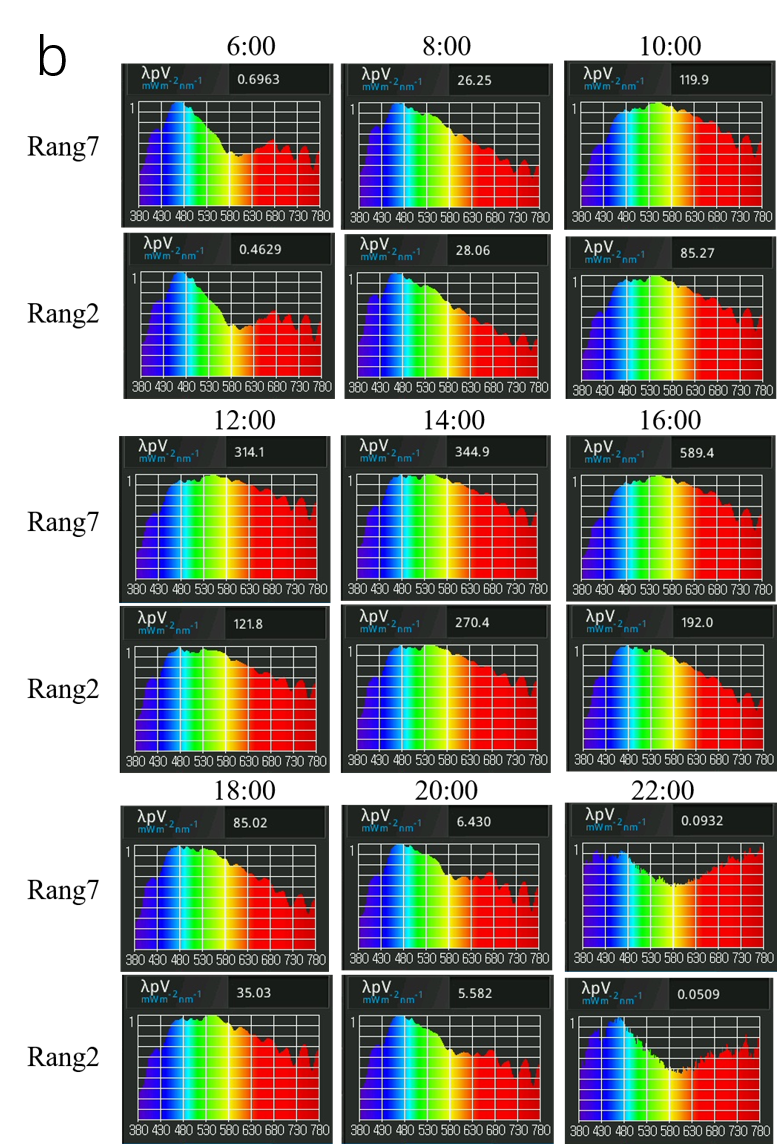


Supplementary Fig. S2: light response curve of leaf gas exchange in comparison between range 2 and range 7 leaf under white light supplementation (a-d) and far-red light supplementation (e-h).


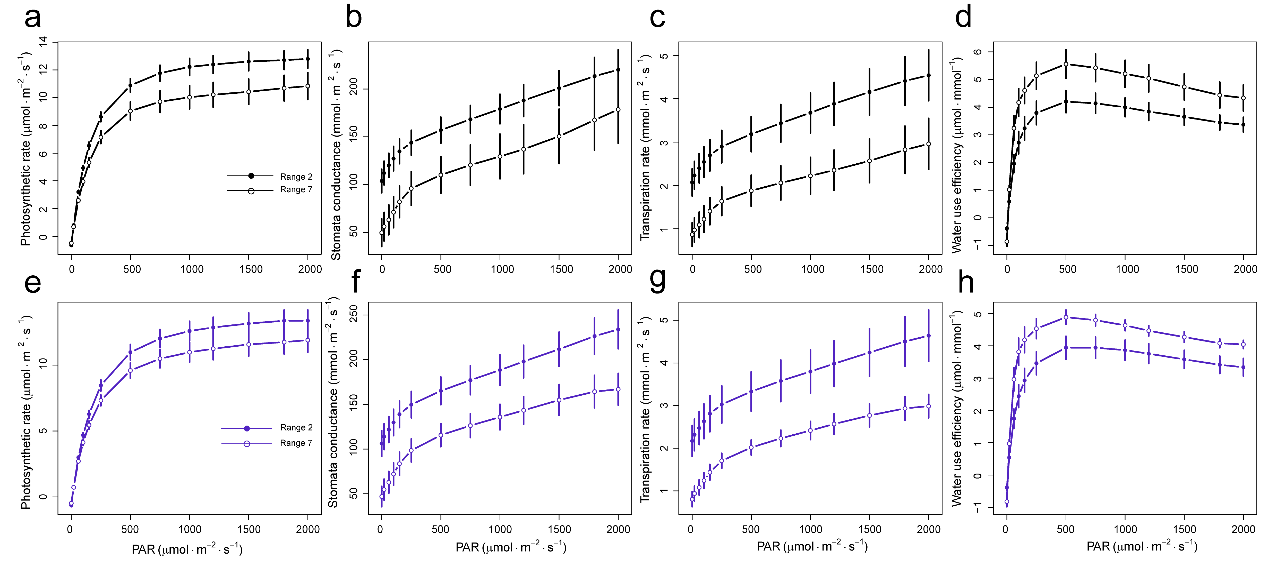


Supplementary Fig. S3**:** GO functional enrichment of DEGs in stems (a) and roots (b).

**a**


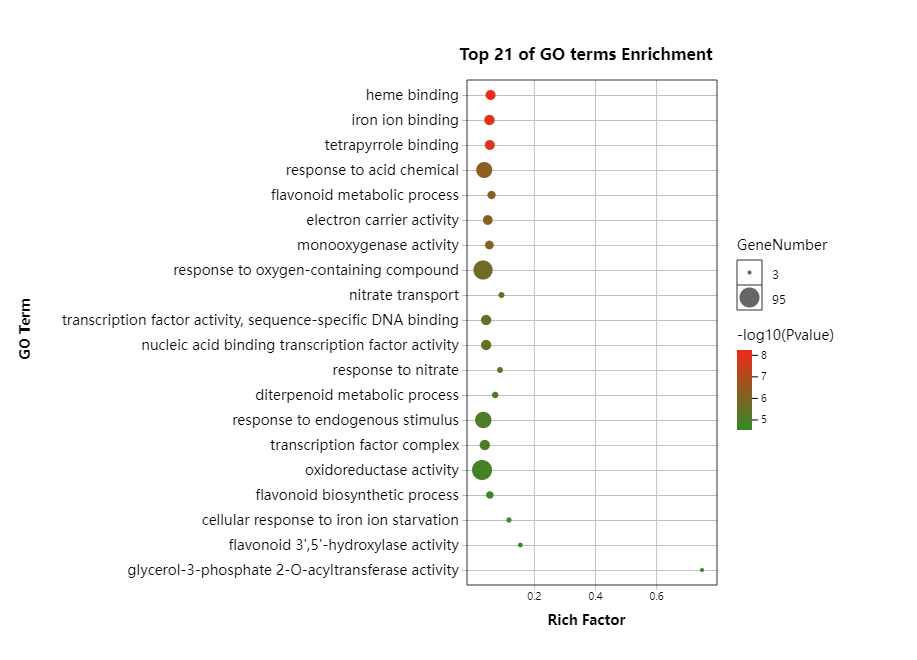


b


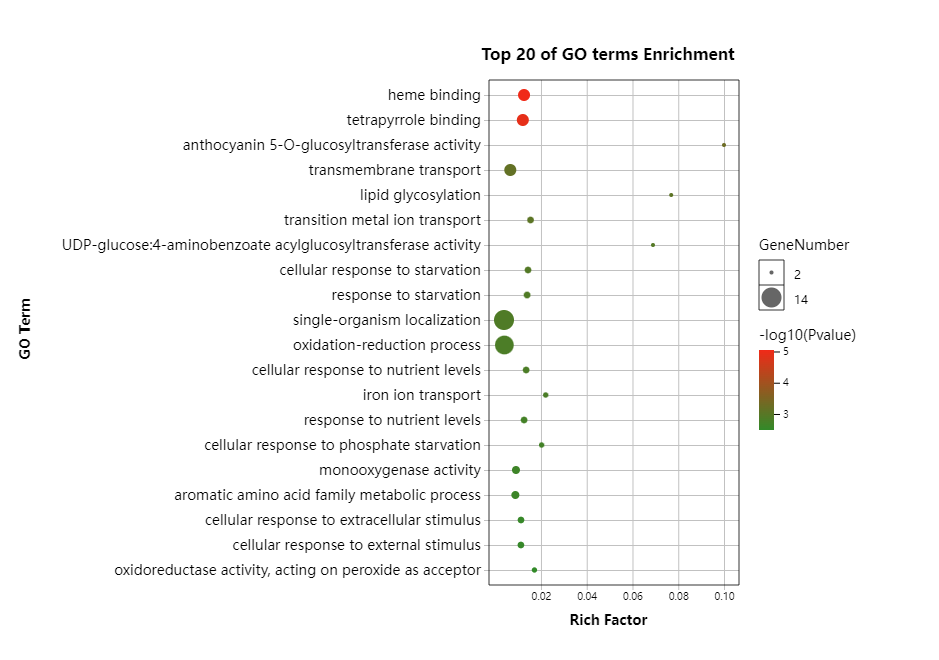


Supplementary Fig. S4**:** GO functional enrichment of the top 50 up- and down-regulated DEGs in leaves (a: downregulated, b: upregulated), stems (c: downregulated, d: upregulated) and roots (e: downregulated, f: upregulated).


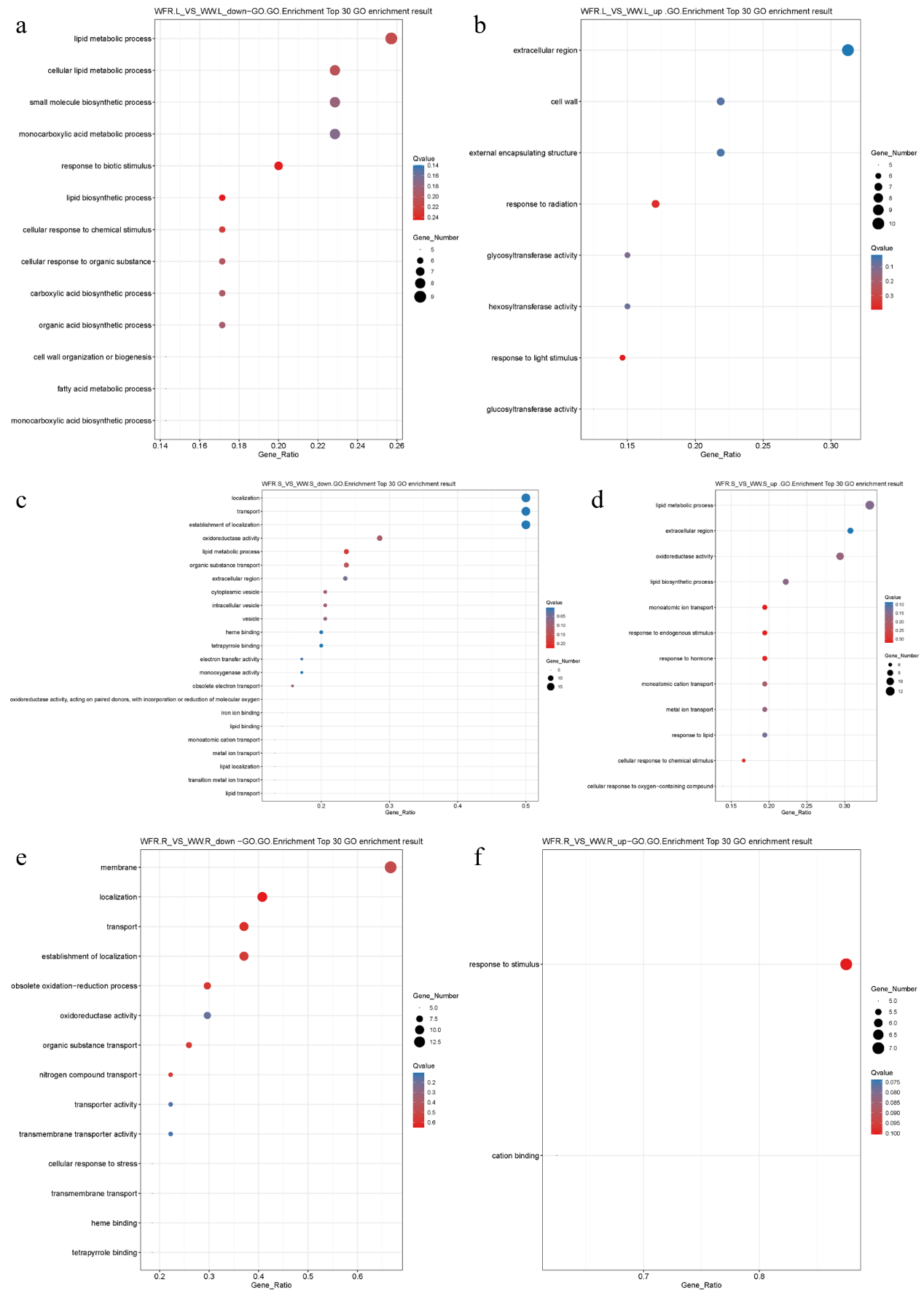


Supplementary Fig. S5: Expression of genes related to redox balance.


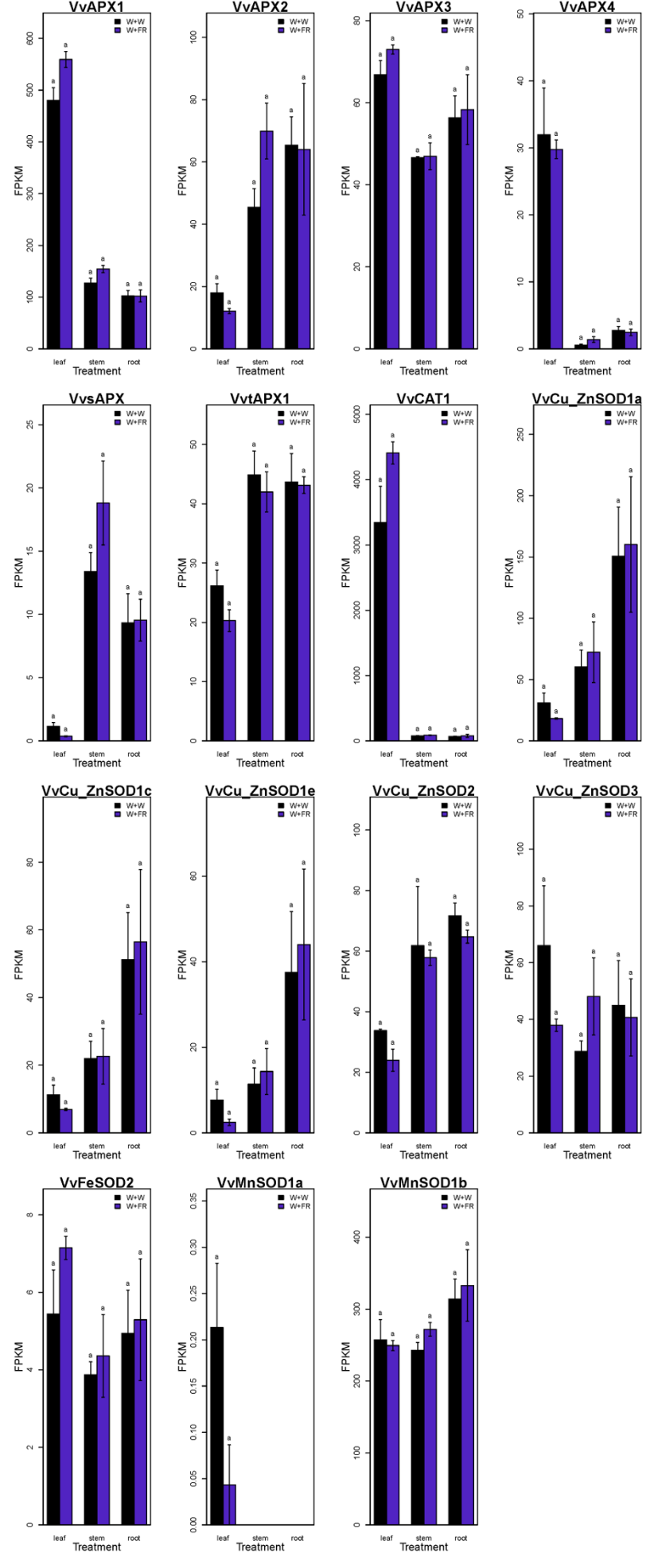


Supplementary Fig. S6: Gene annotation in correlation network in leaves(a), correlation plot between trait and DEGs in leaves(b).

a: Gene annotation in correlation network in leaves


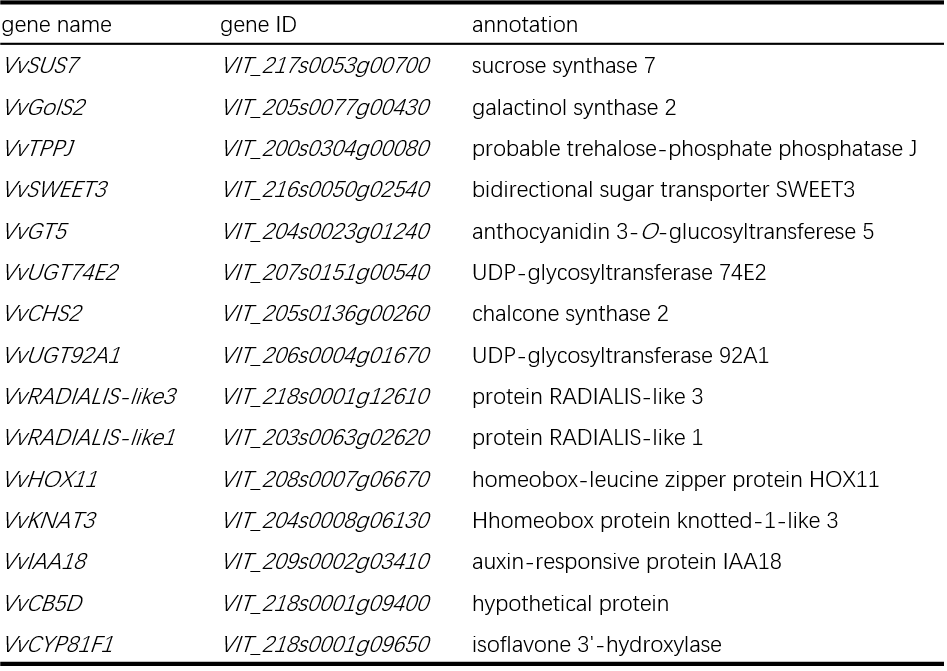


b: Correlation plot between trait and DEGs in leaves


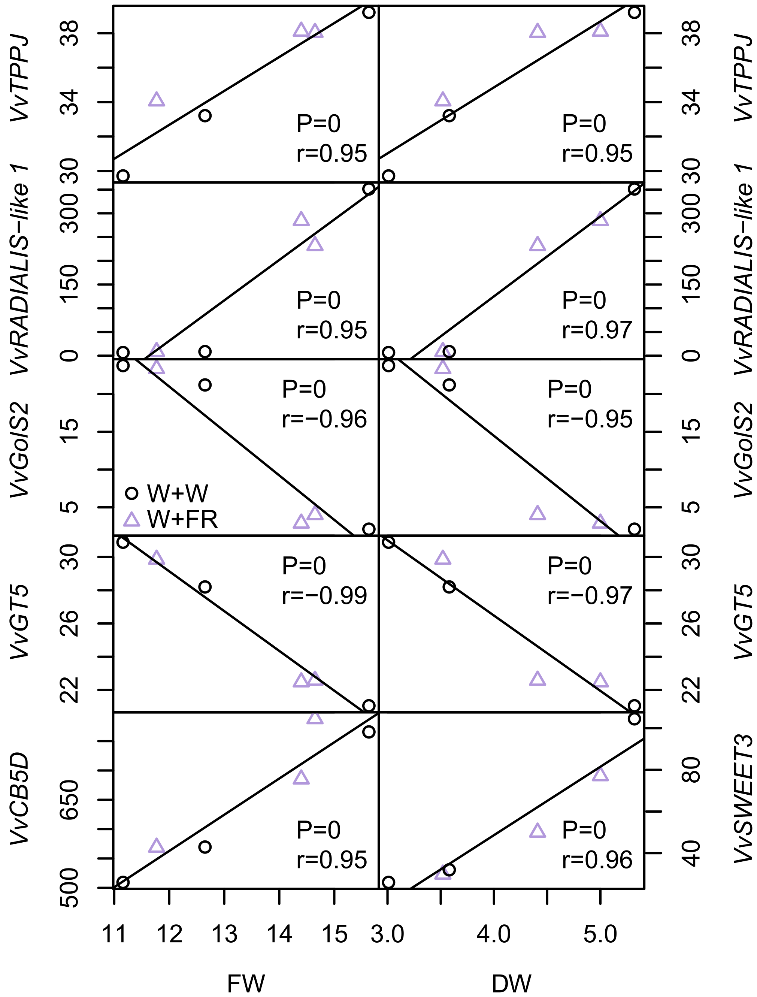


Supplementary Fig. S7: Correlation network between traits and DEGs in stems.


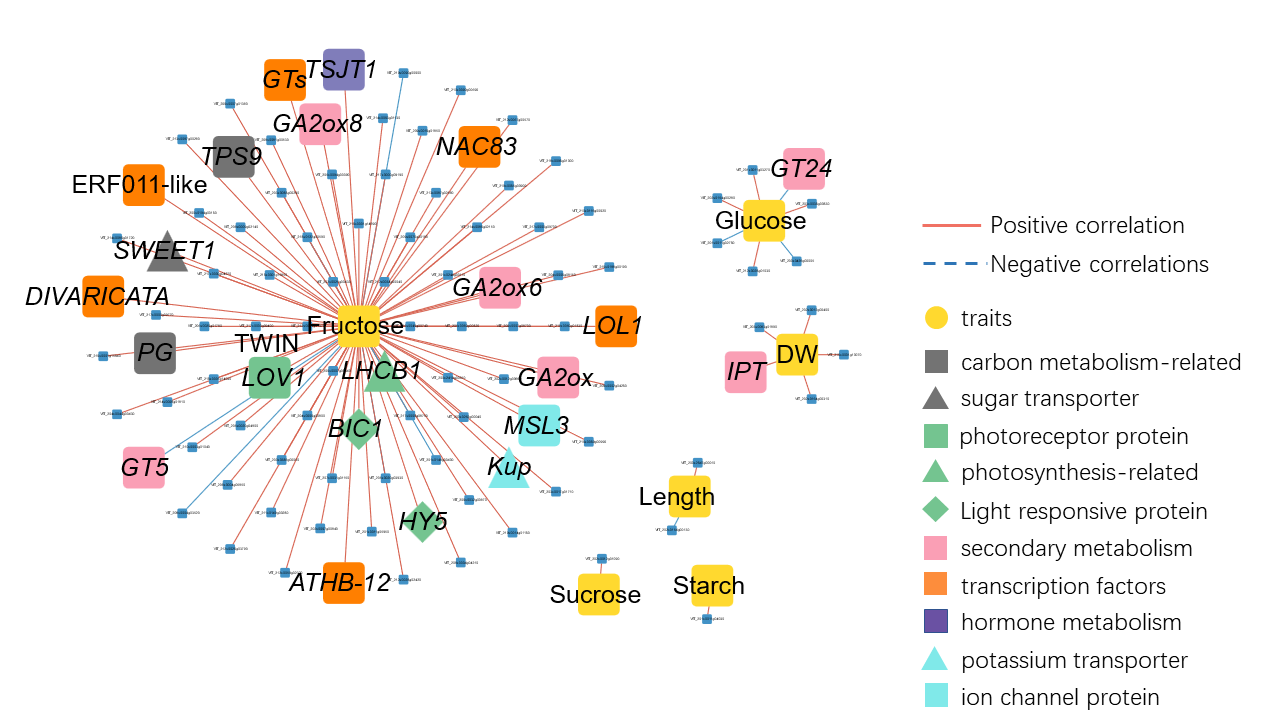

Supplement: Supplementary file 1 — Supplementary Material 1 [file 12870_2024_4870_MOESM1_ESM.docx]
